# Brain mechanisms of automated conflict avoidance simulator supervision

**DOI:** 10.1111/psyp.14171

**Published:** 2022-09-15

**Authors:** Bertille Somon, Aurélie Campagne, Arnaud Delorme, Bruno Berberian

**Affiliations:** ^1^ Département d'Ingénierie Cognitive et Neurosciences Appliquées Office National d'Etudes et de Recherches Aérospatiales Salon‐de‐Provence France; ^2^ LPNC Univ. Grenoble Alpes, Univ. Savoie Mont Blanc, CNRS Grenoble France; ^3^ Swartz Center for Computational Neuroscience University of California San Diego La Jolla California USA; ^4^ Centre de recherche Cerveau et Cognition Université de Toulouse Toulouse France

**Keywords:** automated system, cluster‐based permutation test, EEG, ERP, performance monitoring, time‐frequency analyses

## Abstract

Supervision of automated systems is an ubiquitous aspect of most of our everyday life activities which is even more necessary in high risk industries (aeronautics, power plants, etc.). Performance monitoring related to our own error making has been widely studied. Here we propose to assess the neurofunctional correlates of system error detection. We used an aviation‐based conflict avoidance simulator with a 40% error‐rate and recorded the electroencephalographic activity of participants while they were supervising it. Neural dynamics related to the supervision of system's correct and erroneous responses were assessed in the time and time‐frequency domains to address the dynamics of the error detection process in this environment. Two levels of perceptual difficulty were introduced to assess their effect on system's error detection‐related evoked activity. Using a robust cluster‐based permutation test, we observed a lower widespread evoked activity in the time domain for errors compared to correct responses detection, as well as a higher theta‐band activity in the time‐frequency domain dissociating the detection of erroneous from that of correct system responses. We also showed a significant effect of difficulty on time‐domain evoked activity, and of the phase of the experiment on spectral activity: a decrease in early theta and alpha at the end of the experiment, as well as interaction effects in theta and alpha frequency bands. These results improve our understanding of the brain dynamics of performance monitoring activity in closer‐to‐real‐life settings and are a promising avenue for the detection of error‐related components in ecological and dynamic tasks.

## Abstract 2

Although efficient automated system supervision is a critical issue in high risk environments, error monitoring is mostly characterized for self‐performance. Using a dynamic supervision task, our study points out the similarities between both cognitive processes by assessing the evolution of error monitoring activities and their modification with time on task, also extending previous system error detection results based on time‐frequency patterns.

## 1 INTRODUCTION

The introduction of automation technology in our everyday life has profoundly modified our interactions with the world surrounding us, by having operators performing higher order cognitive tasks whereas the automated systems take over the lower order ones (Berberian, Somon, et al., 2017). Whether in our car, at work or at home, we are now accustomed to interacting with automated systems on a daily basis. It is especially the case for people working in high‐risk domains such as the aeronautics or the nuclear fields, where most processes are highly automated and highly reliable. In this context, operators have moved from “operating”, per se, to supervising these automated systems. Already, in 1983, Bainbridge mentioned: “There are two general categories of task left for an operator in an automated system […] to monitor […] or to take over.” (Bainbridge, 1983) Several consequences of this change in operators' function have been described in the literature. Consequences include a decreased attention and vigilance, a loss of situation awareness, and a decreased motor ability, amongst others (Berberian, Gouraud, et al., 2017; Berberian, Somon, et al., 2017). More particularly, these changes often result in a reduced ability to detect system errors when they occur and to take over when necessary (Endsley & Kiris, 1995). These issues, coined the Out‐Of‐The‐Loop performance problem, have resulted in critical incidents which have had dramatic consequences in terms of casualties and infrastructure costs (e.g., the Three Mile Island power plant accident or the Rio‐Paris flight AF‐447 ending up in the death of its 228 occupants).

Understanding the neurofunctional correlates of system supervision and error monitoring could help prevent these fatalities. In the field of cognitive neurosciences, several studies have tried to elucidate how our brain detects errors and how it corrects them. The error detection process, also called performance monitoring, has raised a lot of attention since the fundamental contribution by Rabbitt (1966) about reaction times related to error detection and correction processes. At the neurophysiological level, performance monitoring has been characterized by a set of early frontocentral and late centroparietal potentials which have been observed in various contexts: (i) after error commission (i.e., the frontocentral negative ERN and centroparietal positive Pe – Falkenstein et al., 1991; Overbeek et al., 2005), and (ii) after feedback observation of an error (i.e., the frontocentral negative FRN and centroparietal positive P300 or RewP – Hajcak et al., 2005; Luu et al., 2003; Proudfit, 2015). The aforementioned components have distinct features. Notably, some of them have been associated specifically to the valence of the outcome (e.g., RewP in reward responsiveness paradigms, where a monetary reward is at stake; Proudfit, 2015), while others not. On the other hand, several authors have suggested a closed link between the ERN‐Pe, FRN‐P300 and N2‐P3 complexes. More precisely they might reflect the performance monitoring process, at different timescales depending on the response selection and execution processes involved (Cavanagh & Frank, 2014; Ullsperger, Fischer, et al., 2014). The role of the valence of the event for the elicitation of these components is still a matter of debate, but many studies settle on a valence‐free expectedness‐based activation of error‐related components (Alexander & Brown, 2011; Pezzetta et al., 2018).

Regarding spectral data, performance monitoring activity has also been linked to specific frequencies: notably frontal midline theta oscillations (FMT) increase associated with cognitive control and evoked by response‐related (Cavanagh & Frank, 2014) as well as feedback‐related (Novikov et al., 2017) mechanisms; but also post‐error alpha suppression in posterior areas (Carp & Compton, 2009; van Driel et al., 2012) associated to attentional adjustments, or attentional enhancement during feedback expectation (Pornpattananangkul & Nusslock, 2016). This post‐error alpha suppression has been demonstrated to vary according to several task parameters such as motivation (Compton et al., 2014) or the type of error (van Driel et al., 2012), but is generally attributed to attentional “reorienting”.

Recent electrophysiological studies have tried to assess error detection during system and human agent supervision (for a review, see Somon et al., 2017). They revealed the emergence of the same kind of event‐related potentials triggered by observing another agent's (human or system) error, taking the shape of an early frontocentral oERN and later centroparietal oPe or an N2 followed by a P3 (Chavarriaga et al., 2014; Somon et al., 2019b; Weller et al., 2018). However, these studies also showed a decrease of the P300 when the observed error is performed by a system in comparison to a human agent (Somon et al., 2019b). Based on the literature, this result can be related to the role of psychosocial parameters (interpersonal similarity – Carp et al., 2009 – empathy – Cracco et al., 2016 – intentionality – Desmet & Brass, 2015 – etc.). Notably, complacency towards automated systems can lead to a lower supervision activity when interacting with them (Parasuraman et al., 1993), which is reflected by a decrease of the P300 activity related to information processing. Complacency is a well‐known precursor of supervision or monitoring decrements. Studying the brain activity related to error detection during everyday‐life‐like automated system supervision would help understand how monitoring difficulties manifest themselves at the neurophysiological level. Nevertheless, most studies about the neurophysiological correlates of performance monitoring are performed in very standardized lab conditions with lab‐oriented protocols and stimuli thus preventing most supervision difficulties to emerge.

In an attempt to decipher the effect of response probability from response type on the performance monitoring brain response during system supervision, and thus determine the role of surprise in the theoretical frame of action observation (Desmet & Brass, 2015), Pezzetta et al. (2018) performed a first‐person perspective virtual reality‐based error monitoring study where the error rate of the avatar's action was 70%. They performed a dynamic task, leading them to study not only ERPs associated to correct and erroneous responses, but also spectral power and time‐frequency measures. Their analyses focused on the FCz and POz electrodes but showed an early FCz‐located greater theta power and alpha power for errors, as well as POz‐located alpha suppression for correct responses compared to avatar errors. Interestingly, they demonstrated that these activities were not related, or at least not solely, to the novelty and surprise triggered by infrequent trials, but confirm the role of theta power in intended goal violation even for continuous dynamic conditions, and alpha power in reorientation of attention. This result is in line with previous literature showing that alpha desynchronization translates the recruitment of attentional resources for task‐relevant events (Klimesch, 2012). However, other studies have also suggested that low‐band (<10 Hz) alpha desynchronization could reflect general task demand and attention as a non‐task specific and widespread brain activity (Gevins et al., 1997; Klimesch, 1999).

The aim of the present study is to determine the neurofunctional correlates of automated system supervision in a more dynamic and applied scenario and verify whether the correlates observed in lab tasks (i.e., an early oERN followed by an oPe, or a more general N2 followed by a P3 in flanker tasks for example) can be observed in this type of more complex situations. A second objective aims at characterizing the evolution of this cerebral activity over time, more precisely the differences in supervision activity that can be observed between the beginning and the end of the supervision task. Indeed, in long‐lasting tasks with repetitive trials and stimuli, participants often bear behavioral performances decrements (Smallwood & Schooler, 2015). It is even more pronounced in supervision tasks. Notably, it has been demonstrated that mind‐wandering (i.e., “…the mind's tendency to engage in thoughts unrelated to the here and now.”; Gouraud et al., 2018) frequency increases after only 20 min spent on an automated system supervision task, and is associated with decreased performances in terms of reaction times (higher and more variable) and accuracy (Bastian & Sackur, 2013; Kam et al., 2012; Lorist et al., 2000). During system supervision, these difficulties usually manifest through a decrease in error detection, but also at the electrophysiological level through decreases of the amplitude of several event‐related potentials (Lorist et al., 2000; Smallwood et al., 2008) and an increase in parieto‐occipital alpha frequency power (Borghini et al., 2014; Campagne et al., 2004; van Driel et al., 2012). Finally, it has been proposed a role of alpha frequency in general attention processes (whether through idling as was initially thought – Klimesch, 1997 – or as more recently hypothesized through inhibition – Klimesch et al., 2007), especially the widespread lower alpha sub‐band ([8‐10]Hz; Gevins et al., 1997).

Here, we recorded electroencephalograms and behavioral data on 18 participants who were asked to supervise a home‐made conflict avoidance simulator (the LIPS – Laboratoire d'intéraction pilote système; Gouraud et al., 2018; Le Goff et al., 2018). Two levels of difficulty were considered to determine how task complexity may impact system monitoring. Likewise, to investigate how this decision‐making process emerges within trials but also can vary during long‐term supervision and across‐time, we looked at the effect of time on task, by comparing the error monitoring activity in the time and time‐frequency domains at the beginning and at the end of the experiment for both errors and correct responses detection. Concerning the overall performance monitoring activity, we hypothesized that the event‐related activity linked to the detection of a system error would be higher in absolute amplitude than the one related to correct response observation in accordance with what is observed during system supervision tasks using more basic and classical stimuli. Specifically, we expected to observe a greater frontocentral negative ERP (oERN or N2; Pezzetta et al., 2018) – given its theoretical role in the detection of divergence from the intended goal – but also a greater centroparietal positive potential (oPe or P300) – given its role in attention reorienting and information extraction – during the detection of system errors compared to correct system responses (Somon et al., 2019b; Weller et al., 2018). In the frequency domain, performance monitoring should take the form of a mid‐frontal theta activity increase for errors, most likely non‐phase‐locked and unlinked to the oERN (Cavanagh & Frank, 2014; Pezzetta et al., 2018; Ullsperger, et al., 2014). In addition, an alpha band activity is expected which may manifest either through: (i) fronto‐central post‐error alpha suppression, as observed after error execution (Carp et al., 2009; van Driel et al., 2012); or (ii) more likely through greater frontal alpha activity for errors as well as parietal alpha suppression after correct responses, as demonstrated in more dynamic error observation tasks (Pezzetta et al., 2018). We also made the hypothesis that these components would be impacted by task difficulty and, thus, an increase in perceptual difficulty would decrease the amplitude of the centro‐parietal positive one, as already observed for error commission and/or observation (Gehring et al., 2011; Somon et al., 2019a; Van der Borght et al., 2016). Regarding the effect of time on task on observational performance monitoring activity, it is expected that complacency leads to the reduction of the overall amplitude of the ERP complex. Finally, we predicted that time on task, independently of the system's response accuracy, would trigger an overall increase of the alpha band activity as it has been demonstrated on several occasions (Borghini et al., 2014; Campagne et al., 2004; Craig et al., 2012).

## 2 METHOD

This research was approved by a local ethics committee (Comité d'Ethique pour les recherches non interventionnelles de Grenoble, n°IRB00010290‐2017‐07‐04‐20‐CERNI_AvisConsultatif‐2017‐06‐13‐04) and conducted according to the principles expressed in the Declaration of Helsinki.

### 2.1 Participants

Based on power analyses performed on previous data assessing performance monitoring during supervision (suggesting a sample size of 14 participants to detect error‐related spectral components during system supervision with *α* = 0.05, the power 1‐*β* = 0.8 and effect size $\eta_{p}^{2}$ = 0.45; Pezzetta et al., 2018) and on sample size typically described in this research domain (including between 15 and 20 participants; van Driel et al., 2012), twenty healthy right‐handed participants (7 women; 27.75 years ± 1.42 years) were recruited for this experiment. All the participants were naïve to the task. They had normal or corrected‐to‐normal vision and hearing, were free of neurological or psychiatric disorders and were not under any medication. The volunteering participants signed a written informed consent and received a financial compensation (30€ in total) for taking part to the experiment. Two participants were removed from data analysis due to bad EEG signal quality (more than one fourth of the data had to be rejected) resulting in a total of 18 participants included in the analyses reported in the results section.

### 2.2 Experimental task and procedure

#### 2.2.1 Stimuli

**FIGURE 1:**
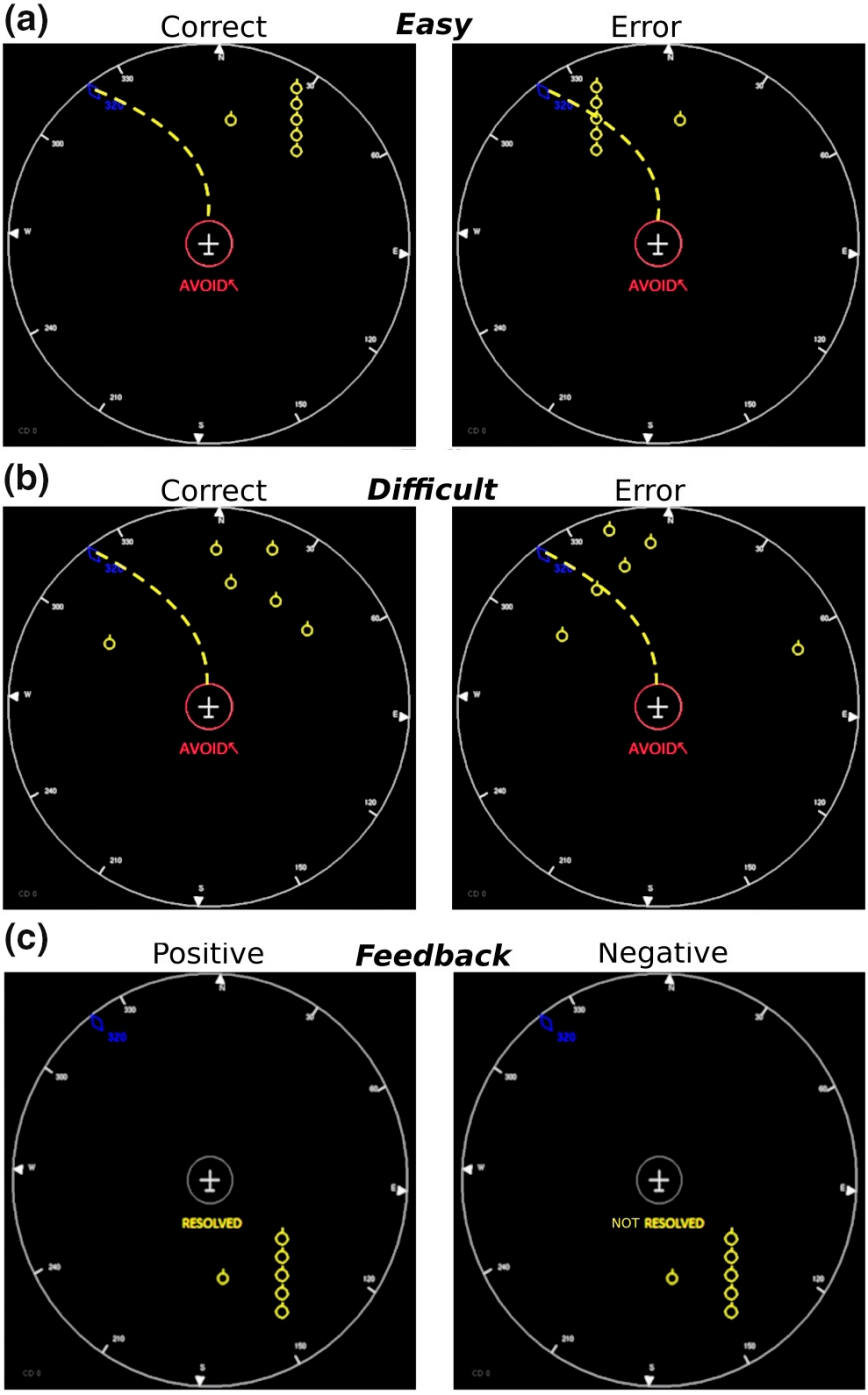
Examples illustrating the different types of trials across experimental conditions. Description of the stimuli presented for responses in easy (a) and difficult (b) conditions, and of the feedback stimuli (c) in the Pilot‐System Interaction Lab experiment. The left side illustrates stimuli for correct response/positive feedback and the right side for erroneous response/negative feedback. Participants had to determine the accuracy of responses according to the distribution of the primary and secondary obstacles. Yellow dotted lines illustrate the flying path taken by the aircraft and are provided for information but were not shown during the experiment.

This experiment consisted in the supervision of an obstacle avoidance simulator (The Pilot‐System Interaction Lab—LIPS; Gouraud et al., 2018; Le Goff et al., 2018) with various levels of difficulty. This simulator took the shape of an aircraft centrally located in a radar zone, in the middle of a screen. The aircraft was moving at a constant speed of 200 m.s^−1^ and was displayed in white onto a black 19‐in CRT monitor (with a 1024x768 pixels resolution and a 100‐Hz refresh rate) located 46 cm away from the participant in an unlit room. Two types of obstacles could appear in a predefined order in the radar zone during the experiment. Both consisted of yellow circles, which were either located on the aircraft trajectory (primary obstacles) or on either side of the aircraft trajectory (secondary obstacles; see Figure 1a).

The arrangement of the secondary obstacles in the radar zone introduced two levels of difficulty within the task: an easy condition for which the secondary obstacles were aligned one above the other on either the right or left side of the screen, and a difficult condition for which the secondary obstacles were randomly dispersed within the upper part of the screen and verified one by one by the experimenter to ensure their accuracy and detectability (see Figure 1a,b).

For each avoidance, a stimulus was displayed by the simulator to inform the participant of the direction chosen by the aircraft to avoid the primary obstacle. This system's response consisted in the word “AVOID” in red along with an arrow indicating either the right (↗) or the left (↖) direction. After each avoidance, two types of feedback expressing the result of the avoidance were also provided to the participant: a positive feedback showing that the aircraft avoided all the obstacles successfully or a negative feedback indicating that the avoidance was not successful (respectively “RESOLVED” or “NOT RESOLVED” written in yellow below the aircraft; see Figure 1c). Overall, four types of trials could be provided to the participant according to the accuracy (correct vs. erroneous avoidance) and the difficulty (easy vs. difficult condition). They are presented in Figure 1a,b.

#### 2.2.2 Procedure

During the experiment, participants had to supervise the obstacle avoidance simulator described above. In this context, the forward‐flying aircraft took the decision to avoid the primary obstacles appearing on its path by a right or left turn. The selected direction of the avoidance maneuver was provided to the participant and was called system's response in the rest of the document. The participant had to assess whether this response was correct or erroneous according to the context surrounding the aircraft (location of secondary obstacles, see Figure 1a,b). Participants' responses were recorded with a computer mouse on which the two buttons were assigned to either the “Correct” or “Error” assessment (response mapping counterbalanced across participants).

The experiment was divided into two experimental sessions taking place on two different days. Every experimental session included six experimental blocks: three in the easy condition and three in the difficult condition completing a total of six blocks for each level of difficulty over the 2 days of experimentation. The order of the blocks was randomized for every participant, and each block was followed by a break. Every block included 25 trials, 40% of which were erroneous (i.e., 10 trials per block, 5 with a right turn and 5 with a left turn). This high error rate was introduced in order to have enough trials for the following EEG analysis and ensure statistical power. Trials were randomly presented. Every experimental session included a total of 150 trials (75 per difficulty level) and lasted approximately 1h15. In total, over the 2 experimental sessions, every participant performed 300 trials (**120** – 40% – erroneous and 180 correct trials; **150** – 50% – right turns and **150** – 50% – left turns; 150 trials in the easy condition and 150 trials in the difficult condition).

Each trial started with the aircraft displayed in white against a black background at the center of the screen and of the radar zone. Obstacles progressively appeared at the top of the screen: one primary obstacle and five secondary obstacles. When the aircraft was close enough from the primary obstacle the simulator detected the possibility of a collision (roughly 7.45 s before the putative conflict) and sent a message to the participant by displaying “CONFLICT AHEAD”. The simulator then initiated a conflict avoidance mode 200 ms after conflict detection. At that moment, the system's response was provided to the participant indicating the direction taken by the aircraft to avoid the primary obstacle, and the simulation froze.

After a 1‐s delay during the freezing period, the participant had to assess the accuracy of the simulator's decision. The end of this delay was indicated by a change in color of the circle surrounding the aircraft, which turned green. The participant responded by clicking one of the two buttons of the computer mouse: “Correct” or “Error”. The simulation stayed frozen until a response was given.

The role of this freezing period was to ensure that the quantity of information provided to the participant to make a decision was the same for every trial for each participant. Similarly, the delay between the system's response and the response of the participant ensured that the brain activity associated with error‐detection was not disrupted by motor‐related brain activity. Nevertheless, this delay also prevents us from analyzing reaction times as a performance indicator.

**FIGURE 2:**
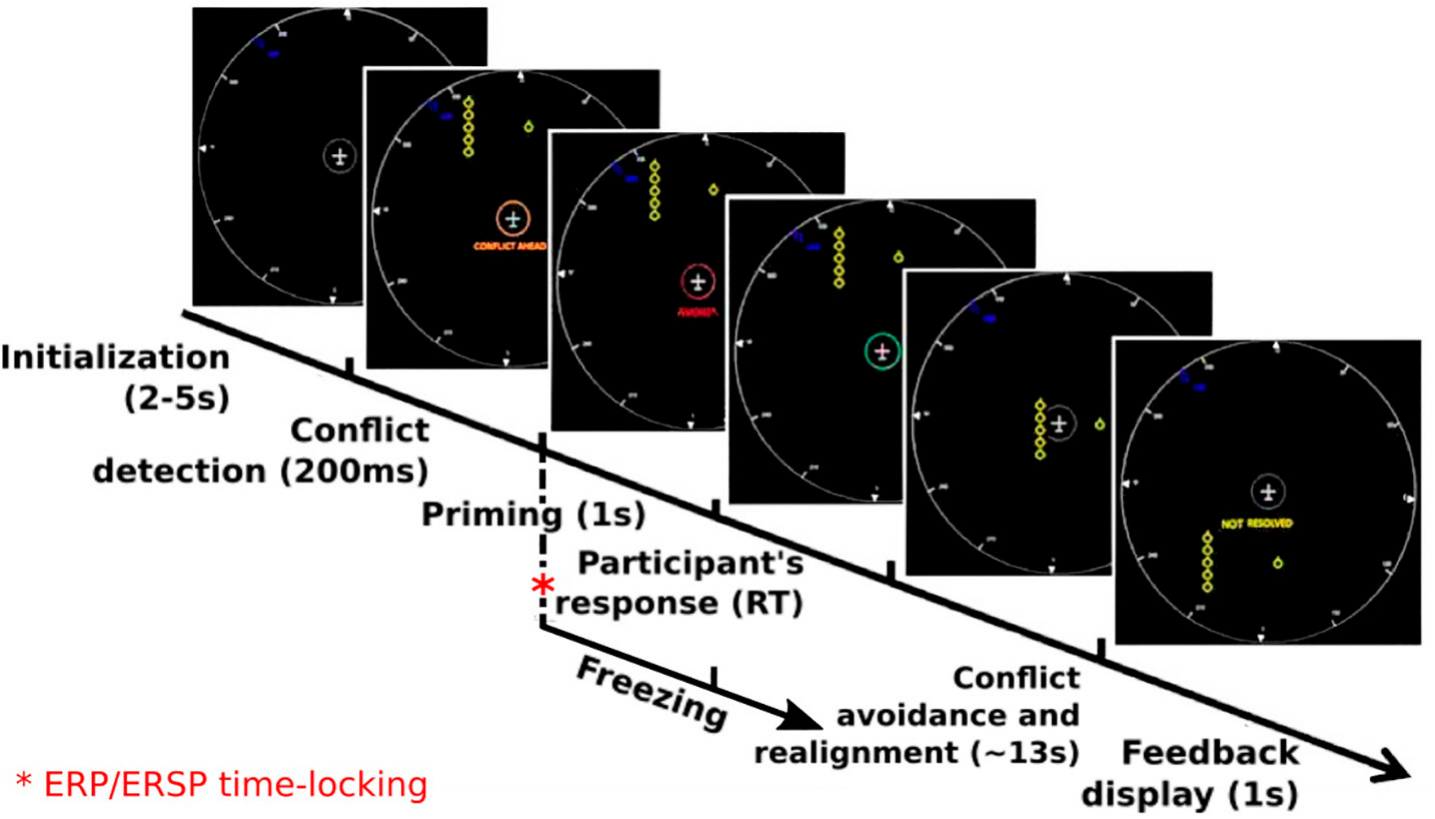
Description of a trial and its time course (case of the easy condition). Every time the aircraft faced a primary obstacle (isolated yellow circle in the pathway), it engaged the conflict avoidance and its safety circle turned orange. The system's response was then provided in red for 1 second and that moment corresponded to the start of the freezing period. It was followed by the safety circle turning green indicating to the participant that he could provide his response on the accuracy of the avoidance according to the spatial location of the secondary obstacles (i.e., the five yellow circle here aligned on the left‐hand side of the screen). When the participant gave his response, freezing stopped and the simulation resumed; the aircraft performed the avoidance and realigned on its initial trajectory. A feedback on the accuracy of the avoidance maneuver was then displayed.

Once the participant had responded, the simulation started again, and the aircraft engaged in the avoidance of the primary obstacle in the direction chosen by the simulator. This avoidance lasted approximately 13 seconds, depending on the disposition of the obstacles. If the aircraft encountered secondary obstacles during its avoidance phase, it created a conflict and thus an erroneous trial. The aircraft then came back to its initial path and the simulator sent a feedback to the participants by indicating whether the avoidance was correct (‘RESOLVED’) or not (‘NOT RESOLVED’). The response of the participant had no impact on the simulation except for unfreezing it, yet the participant could understand this feedback as positive, when his/her response was in accordance with the feedback, or negative when it was not. Finally, the aircraft kept a straight trajectory until new obstacles appeared, triggering another trial. A timeline of a complete trial is provided in Figure 2.

All participants underwent a familiarization phase for each difficulty level before every experimental session corresponding to half a block in each case (12 easy and 12 difficult trials) lasting approximately 10 min. Trials from this familiarization phase were not included in subsequent analyses.

### 2.3 Measure and analysis

#### 2.3.1 Subjective measures

Participants' vigilance and emotional states were acquired at the beginning and at the end of each experimental session respectively through a Karolinska Sleeping Scale (from 1 – Very Awake – to 9 – Very Sleepy) and Likert scales (from 0 – very little – to 10 – very much) assessing calm, stress, joy and boredom. All these variables were analyzed with a two‐way repeated‐measures ANOVA with the moment of the session (Beginning vs. End) and the experimental session (session 1 vs. session 2) as within factors. Mean comparisons were then performed using a Tukey HSD post‐hoc test.

Task difficulty was assessed at the end of every experimental block with a Likert scale going from 0 – Easy – to 9 – Difficult. Task difficulty was statistically analyzed with a paired Student's t‐test according to the level of difficulty of the block (Easy vs. Difficult).

All analyses were performed with Python (v.3.7.10) and the Pingouin library (v.0.4.0), and results were reported as mean ± SEM. Generalized eta squared (*η* ^2^) are provided as a measure of the effect size (Bakeman, 2005; Olejnik & Algina, 2003). Significance level was placed at .05.

#### 2.3.2 Behavioral measures

Reaction times were recorded for each participant and every trial. However, given the imposed delay between the cue and the response, and the infinite time provided to the participant for responding, they were not analyzed in this study.

In addition, participants hit rates was computed for each session and each level of difficulty (Easy vs. Difficult) as the number of correct estimations of the accuracy of the system's response (i.e., whether the system is going to do a correct avoidance or an erroneous one) over the total number of trials in each level of difficulty and selected moment of the experiment (three blocs at the beginning and at the end). Hit rates were compared across participants with two different pairwise t‐tests with Bonferroni correction: (i) with the level of difficulty (Easy vs. Difficult), and (ii) with the moment of the experiment (Beginning vs. End) as within‐subject factors. Analyses were performed with Python (v.3.7.10) and results were reported as mean ± SEM. Generalized eta squared (*η* ^2^) are provided as a measure of the effect size (Bakeman, 2005; Olejnik & Algina, 2003). Significance level was placed at .05.

#### 2.3.3 Electroencephalography

The electroencephalogram (EEG) was continuously recorded using an ActiCAP (Brain Products GmbH) equipped with 64 Ag/AgCl unipolar active electrodes which were positioned according to the extended 10–20 system (Jasper, 1958). The reference and ground electrodes used for EEG data acquisition were positioned on the forehead (respectively AFz and Fpz electrodes). Blinks and eye movements were also monitored using four pure silver electro‐oculography electrodes: two positioned above and below the left eye on the median axis for vertical activities and two at the eyes' outer canthi for horizontal activities. The ground electrode for the EOG was placed on the right earlobe. In addition, participants were instructed to limit blinking and eye‐movements. Signal impedance was kept below 10 kΩ for all electrodes. The signal was amplified using an ActiCHamp™ system (Brain Products, Inc.), digitized at a 24‐bit rate and sampled at 1000 Hz, with a 0.05 μV resolution.

All EEG data analyses were performed using EEGLAB v2019.1 (Delorme & Makeig, 2004) and Fieldtrip (Oostenveld et al., 2011) MATLAB (R2019b) toolboxes (The MathWorks, Inc.). The raw EEG data were re‐referenced offline to the linked mastoids. The signal was segmented into 30s‐epochs that started with trial onset. The signal was then down‐sampled to 500 Hz and band‐pass filtered between 0.5 and 40 Hz (8th order band‐pass Butterworth filter from ERPLAB; Lopez‐Calderon & Luck, 2014). Artifacts related to ocular movements (saccades and blinks) were visually identified and manually rejected after decomposing the data with an Independent Component Analysis (ICA) with the extended infomax algorithm included in EEGLAB. At most four ICs were removed (except for one participant whose eye‐artifacts ICs were always split into two leading to the removal of seven ICs in the first fifteen), selected from the first ten components reordered by variance, according to the recommendations on the EEGLAB and ICLabel wiki. Data were then re‐segmented into 3.5 s‐epochs (−1500 to 2000 ms) time‐locked to the system's response display (see Figure 2). All segments contaminated with muscular activity and/or non‐physiological artifacts were rejected offline after a visual inspection. Data were then baseline‐corrected from −500 to 0 ms.^1^ The long pre‐stimulus time window was selected in order to perform time‐frequency analysis on the data.

EEG data related to system's response were averaged and analyzed in two different ways. First, they were considered according to the accuracy of the system response (Error vs. Correct) and task difficulty (Easy vs. Difficult). Then they were considered according to the accuracy of the response of the system (Error vs. Correct) and the moment of the experiment (Beginning vs. End). For this second analysis, the two first blocks (beginning) and two last blocks (end) of every experimental session were averaged for each participant, independently of task difficulty. For both analyses, trials wrongly classified by the participant (false alarms, omission, etc.) were not considered.

EEG data were analyzed both in the time domain, through the usual event‐related potentials (ERPs), and in the time‐frequency domain, through the event‐related spectral perturbations (ERSPs). Finally, the variations in the EEG activity across trials were assessed with ERP‐images and inter‐trial coherence (ITC). For all statistical analyses, the significance level was set at α = .05 after correction for multiple comparison using FDR.

##### ERP analysis

Mean ERPs were averaged in the time domain for the four conditions for the first (*accuracy* x *task* *difficulty*) and second (*accuracy* x *moment of experiment*) analysis. Significant differences in ERPs between the various conditions for each analysis were assessed using a cluster‐based permutation test with the Fieldtrip toolbox of MATLAB. This test is based on the cluster mass test (Maris & Oostenveld, 2007; Oostenveld et al., 2011) and identifies spatio‐temporal clusters presenting a significant difference in ERP data between the conditions in a given time period. This method is now commonly used in EEG experiments (Sassenhagen & Draschkow, 2019; Somon et al., 2019b) and has for main benefit to require no a priori on the statistical distribution of the data. Likewise, there is no need to predefine the localization or the time‐window for the ERP analyses. However, it has to be noted that the spatio‐temporal course of the identified clusters by the test, i.e., onset, offset and spatial composition of the clusters, remains approximate and is provided for information (Sassenhagen & Draschkow, 2019). The experimental conditions were compared separately two‐by‐two with dependent samples tests and the Monte Carlo method to compute the significance probability.

##### ERSP analysis

EEG data were also analyzed in the time‐frequency domain through Event‐Related Spectral Perturbations (ERSP) analyses. This type of analysis makes it possible to uncover evoked as well as induced oscillations by studying their power variation across time for each frequency band. For each electrode, ERSPs were obtained through a complex Morlet wavelet transformation applied for every trial. For an optimal frequency resolution, wavelet cycles were fixed at 3 cycles at the lowest frequency (3 Hz) and increased linearly until they reached a maximum of 8 cycles for higher frequencies (40 Hz). Time‐frequency data across trials were then averaged per condition for each participant and baseline corrected through dB‐normalization. The literature review as well as visual inspection of the time‐frequency maps revealed activity in the lower alpha (α: 8–10 Hz) and theta (θ: 4‐8 Hz) frequency bands, either related to performance monitoring processes or attentional resources recruitment.

Due to initial high dimensionality of ERSP data (assessing power spectral density at each channel × time × frequency point) in each targeted condition, our first step was to reduce this dimensionality for statistical analysis. To this aim, time‐frequency data were first subject to a statistical non‐parametric permutation test with FDR correction for multiple comparisons (as implemented in EEGLAB) according to the same conditions as in the ERPs analyses – i.e. *accuracy* × *difficulty* for the first analysis and *accuracy* × *moment of experiment* for the second one – across the various frequencies (0‐40 Hz) at the FCz electrode, which is a usual site for performance monitoring activity. This aimed at approximating the time and frequency window allowing to observe differences between the various conditions. The absence of significant difference at this specific electrode led us to base our assumptions on (i) the literature review, and (ii) visual inspection of time‐frequency data, and further restrict the analysis to frequency bands of interest: theta – 4 to 8 Hz (Cavanagh & Frank, 2014; Pezzetta et al., 2018) – and lower alpha – 8 to 10 Hz (Gevins et al., 1997; Klimesch et al., 2007). The spatio‐temporal distribution of the mean spectral power in these frequency bands were statistically analyzed by considering the conditions of each analysis as within‐subject factors. For this we averaged the power spectral density over the lower alpha and over the theta frequency bands for each trial for every participant and submitted these time courses of mean spectral power to a dependent samples cluster‐based permutation test used previously for the ERP analysis. Clusters differentiating significantly the various experimental conditions two‐by‐two and their spatio‐temporal characteristics are reported in the results section. They were compared with dependent samples tests and the Monte Carlo method to compute the significance probability.

Finally, in order to assess the temporal dynamics of system response evaluation in this task and variance across trials, trial‐by‐trial time‐frequency (ERP‐images under EEGLAB) and inter‐trial phase coherence analyses (ITC) were performed. These analyses were particularly performed in the theta frequency band, which is usually linked to decision making, and at the FCz electrode which is a usual site for performance monitoring activity (FCz was included in the significant clusters obtained in the previous ERP analysis). For this, trials were sorted according to the maximum of the phase time‐locked to the theta activity peak latency. These two analyses allowed to determine and isolate evoked from induced activity. Results for trial‐by‐trial analyses are reported in the Supporting Information, as they showed no significant differences between any of the conditions but can still be informative on the type of activity observed during system supervision.

## 3 RESULTS

### 3.1 Subjective measure

#### 3.1.1 Vigilance

Mean vigilance level was significantly impacted by the moment of the experiment (*F*[1,17] = 51.26, *p* < .005, *η* ^2^ = .44), the experimental session (*F*[1,17] = 6.9, *p* < .05, *η* ^2^ = .07) and the interaction between both (*F*[1,17] = 4.53, *p* < .05, *η* ^2^ = .02). Mean comparisons revealed that participants reported being significantly less vigilant during the first session (5.22 ± 0.27) compared to the second one (4.50 ± 0.26; *p* < .05) but also that they were less vigilant at the end of sessions (6.03 ± 0.30) compared to the beginning (3.69 ± 0.26; *p* < .005). More precisely, interactions revealed that they were less vigilant at the end of the first session (6.56 ± 0.33) compared to the end of the second session (5.50 ± 0.36; *p* < .005), even though there was no difference between the beginning of the two sessions.

#### 3.1.2 Emotions

There was no effect of the experimental session on any of the emotions considered. Nonetheless, boredom and stress were significantly modulated by the moment of the experiment (*F*[1,17] = 50.40, *p* < .005, *η* ^2^ = 0.19 and *F*[1,17] = 4.67, *p* < .05, *η* ^2^ = 0.01 respectively). Mean comparisons revealed that boredom increased significantly (4.11 ± 0.36 vs. 6.28 ± 0.40) while stress decreased significantly (2.22 ± 0.41 vs. 1.69 ± 0.34) from the beginning to the end of the experimental sessions. An interaction effect was observed on happiness between the experimental session and the moment of the experiment (*F*[1,17] = 4.64, *p* < .05, *η* ^2^ = 0.006). Mean comparisons revealed that participants were significantly happier at the end (6.5 + 0.45) compared to the beginning (5.94 ± 0.42) of the second session. Calmness was not impacted by any of the factors.

#### 3.1.3 Difficulty

Participants reported that difficult blocks were significantly more difficult (2.12 ± 0.15) than easy ones (1.07 ± 0.13; *t*[17] = −5.0675, *p* < .005).

### 3.2 Behavioral measures

**FIGURE 3:**
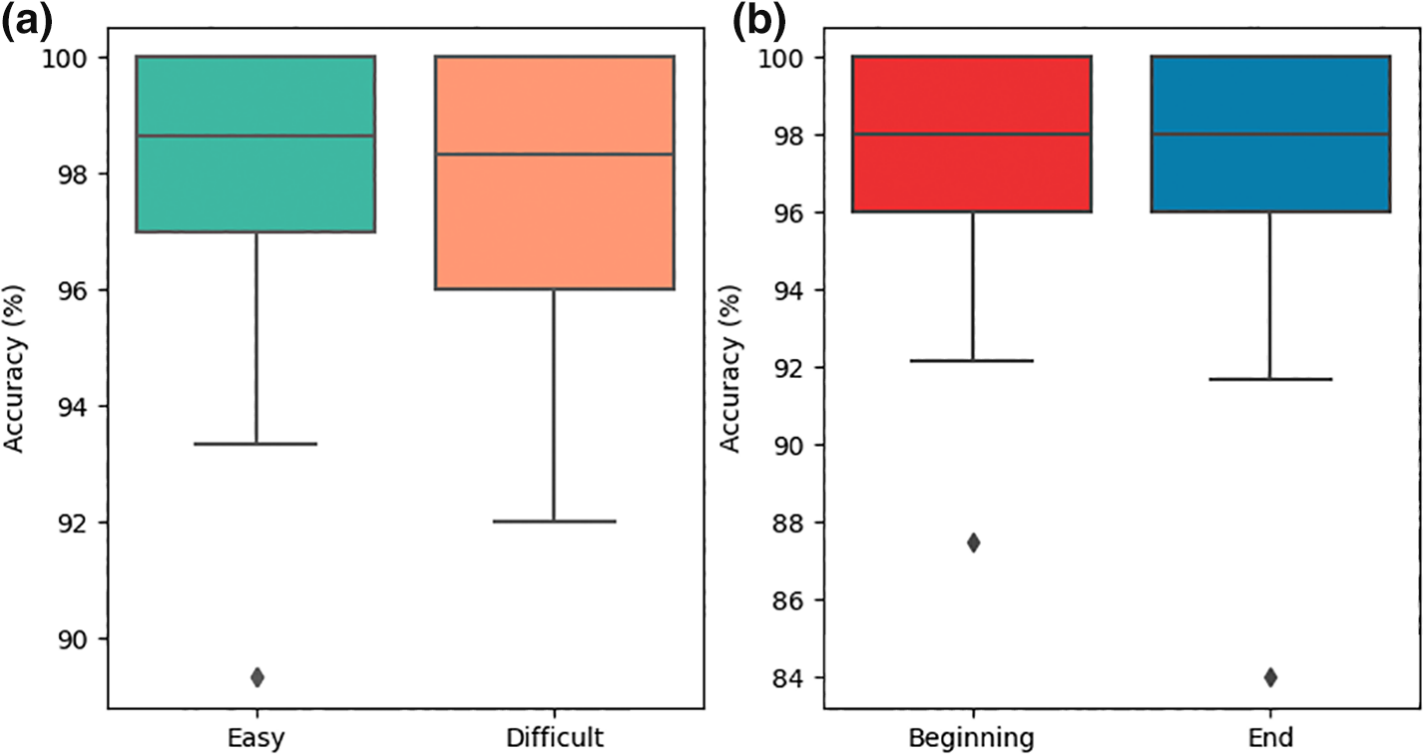
Boxplots of participants' accuracy (%) for the system supervision task according to (a) the difficulty level (Easy – green, left – vs. Difficult – orange, right) and (b) the moment of the experiment (Beginning – red, left – vs. End – blue, right). Boxes show the median (midline) and the (first and third) quartiles of the dataset while the whiskers extend to show the rest of the distribution.

Average participants' hit rate across all conditions was 97.61 ± 0.57%. The pairwise t‐test on the effect of difficulty revealed no effect of the difficulty level on participants' hit rates (easy vs. difficult; *t*[17] = −0.35, *p* = .73; see Figure 3a). Likewise, the pairwise t‐test on the effect of the moment of the experiment did not reveal any statistical effect of the moment of the experiment on participants' hit rates (beginning vs. end; *t*[17] = 0.59, *p* = .57; see Figure 3b).

### 3.3 EEG measures

#### 3.3.1 ERP analysis

##### Analysis according to accuracy and task difficulty

**FIGURE 4:**
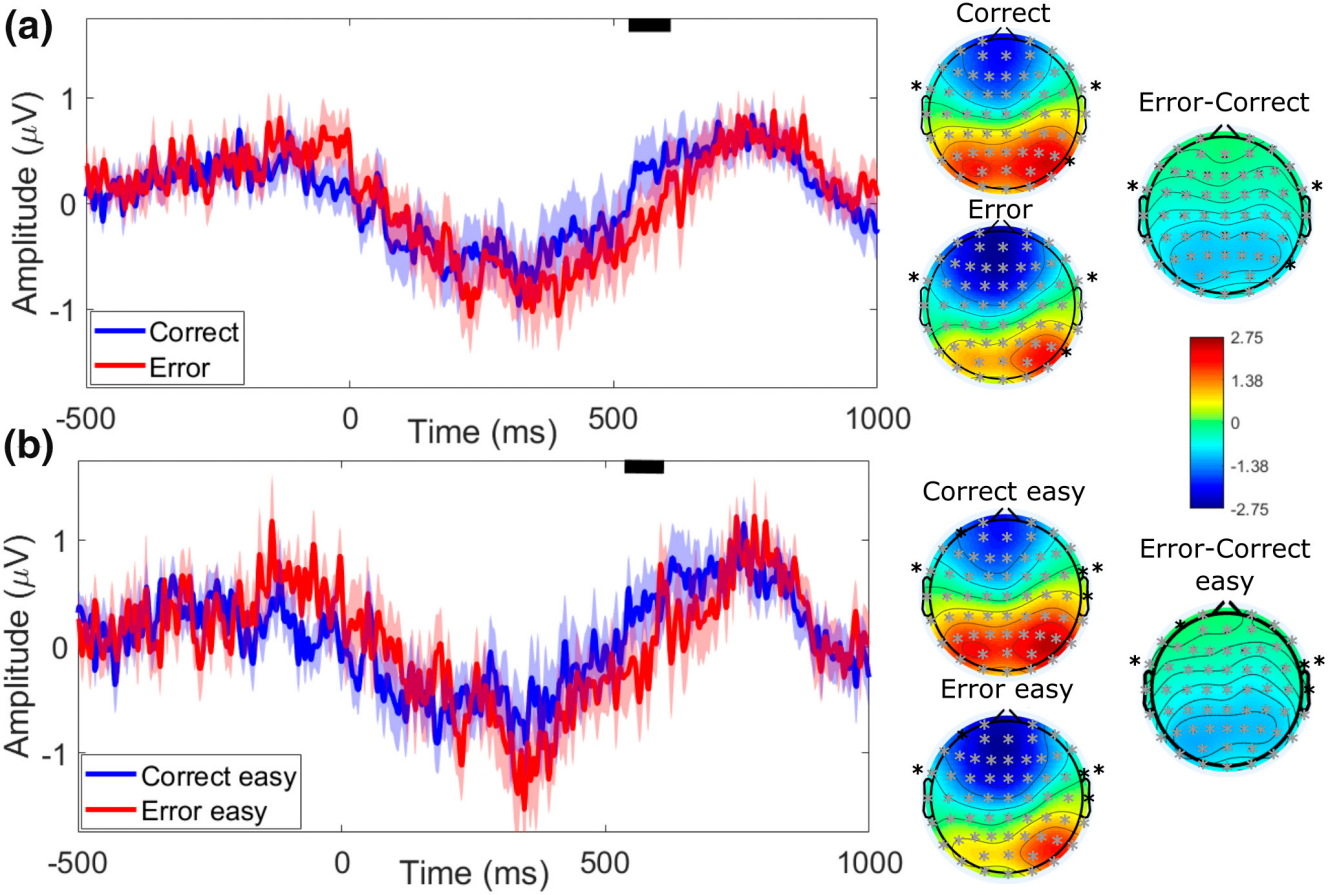
Time course (low‐pass filtered at 30 Hz for display, between −500 and 1000 ms) and topographies of the clusters differentiating significantly the detection of errors (red) and correct responses (blue) of the pilot‐system interaction lab during its supervision (response onset at 0 ms) (a) for both levels of difficulty and (b) for the easy condition only. Black lines show the time‐window during which the clusters of activity are significant. Topographies represent the mean activity of the electrodes included in each cluster (gray crosses) during this significant time‐window for each condition (correct and error – top – and correct easy and error easy – bottom) and for the difference topographies (error vs. correct – top – and error vs. correct for the easy condition only – bottom). Black crosses are the electrodes not included in the significant cluster, i.e., in the averaged topography. The amplitudes of activity represented on the topographies go from positive (max. 2.75 μV in red) to negative (min. ‐2.75 μV in blue) relative to baseline.

The cluster‐based permutation test revealed a significant difference, spread over the whole scalp, ranging from approximately 530 to 610 ms post‐system response (*p* < .01) whose activity amplitude was significantly lower for system error detection compared to correct system responses observation, regardless task difficulty. When separating data according to task difficulty, the permutation test revealed a similar difference discriminating significantly the activities related to accuracy detection of system response (ranging approximately from 540 to 610 ms post‐system response display) in the easy condition only. Here, the amplitude of the system error detection‐related activity was significantly lower than the amplitude of the correct system response detection‐related activity (*p* < .05). These results and their topography are presented in Figure 4.

No significant difference in activity between the easy and difficult conditions was identified, whatever the accuracy of system response.

It must be noted though that the very conservative cluster‐based permutation analysis did not allow us to observe a statistical difference regarding usual performance monitoring negative peaks (N2/oERN) in the conventional [200; 300]ms post‐response observation time‐window. Nevertheless, a further complementary two‐way repeated measures ANOVA at these latencies demonstrated a main effect of accuracy on the amplitude of the most negative peak at the FCz electrode (*F*[1,17] = 10.55, *p* < .005, *η* ^2^ = 0.022) with the amplitude at the peak more negative for error observation (−3.17 ± 0.53 μV) compared to correct response observation (−2.47 ± 0.55 μV).

##### Analysis according to accuracy and moment of the experiment

A cluster‐based permutation test revealed a significant difference of activity for the detection of system errors compared to the detection of correct system responses (*p* < .01). This difference seems similar to the one previously described showing a lower amplitude with the difference spread over the whole scalp similarly to the one of the first analysis (see Figure 4a), except for occipital electrodes which were not discriminant. No effect of the moment of the experiment was observed in the ERP data.

#### 3.3.2 ERSP analysis

##### Analysis according to accuracy and task difficulty

Permutation analysis on time‐frequency data at electrode FCz across all frequencies showed no effect of accuracy or task difficulty on spectral power time‐locked to system response. Nevertheless, visual inspection of the averaged time‐frequency maps across trials per condition showed a predominant performance monitoring related low frequency activity (theta and lower alpha) following system response observation, irrespective of its accuracy.

###### Theta activity (4–8 Hz)

**FIGURE 5:**
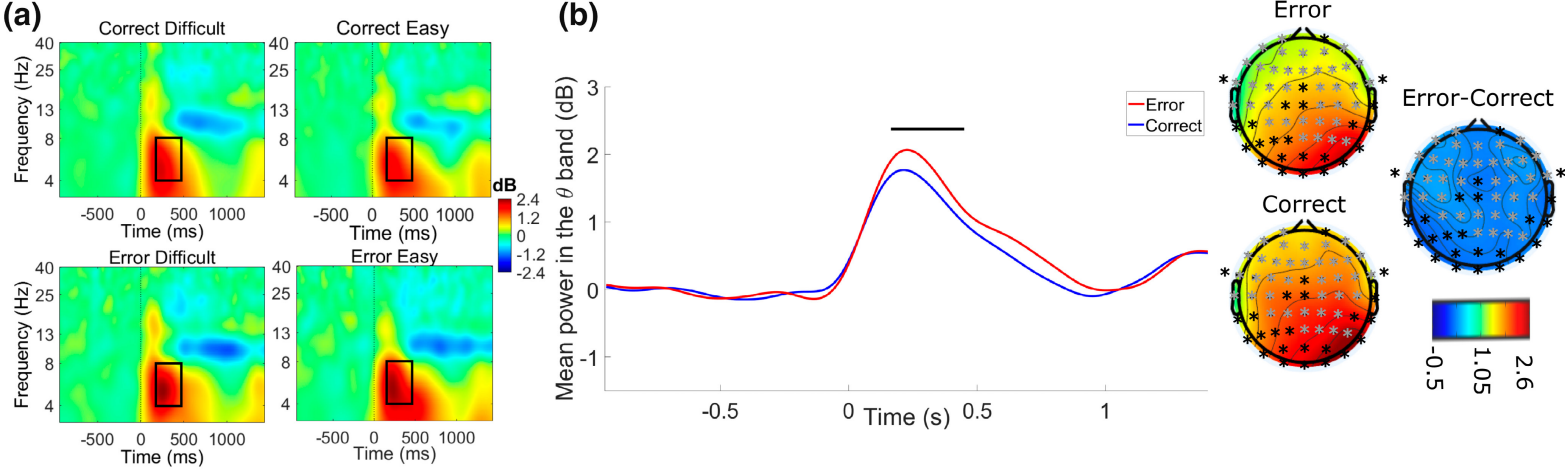
(a) Mean time‐frequency activity time‐locked to system response (0 ms) averaged across the 38‐electrode cluster (gray crosses) in the difficult (left) and easy (right) conditions for correct system responses (top) and system errors (bottom) averaged across participants. Graphs show the power spectral density across trials (between −1 and 1.5 s) for each frequency (1‐40 Hz) according to baseline: Higher values (increases) are in red and lower values (decreases) are in blue. The black rectangle represents the outline of the significant cluster in terms of time and frequency windows. (b) Time course and topography of the difference in theta activity between errors (red line, top topography) and correct responses (blue line, bottom topography) independently of task difficulty. The black line represents the significant time‐window. Topographies show the average theta band power at the 38 electrodes of the cluster (gray crosses) during the significant 160‐450 ms time‐window, and the mean difference topography (right topography) of the theta band activity between errors and correct responses (from −0.5 – blue – to 2.6 dB – red – relative to baseline).

A cluster‐based permutation revealed a significant difference in the theta frequency band (*p* < .05) between system error detection and correct system responses observation. In the 160 to 450 ms frequency range this difference was pronounced on a large number of electrodes, including mainly fronto‐temporo‐parietal electrodes. A higher activity was observed for system error compared to correct system response detection (see Figure 5).

No effect of difficulty or interaction effect with this factor was observed on theta band activity time‐locked to system response.

###### Low alpha activity (8–10 Hz)

No effect of accuracy, difficulty, or interaction effect between both was observed on the mean power of the low alpha activity time‐locked to system response.

##### Analysis according to accuracy and moment of the experiment

**FIGURE 6:**
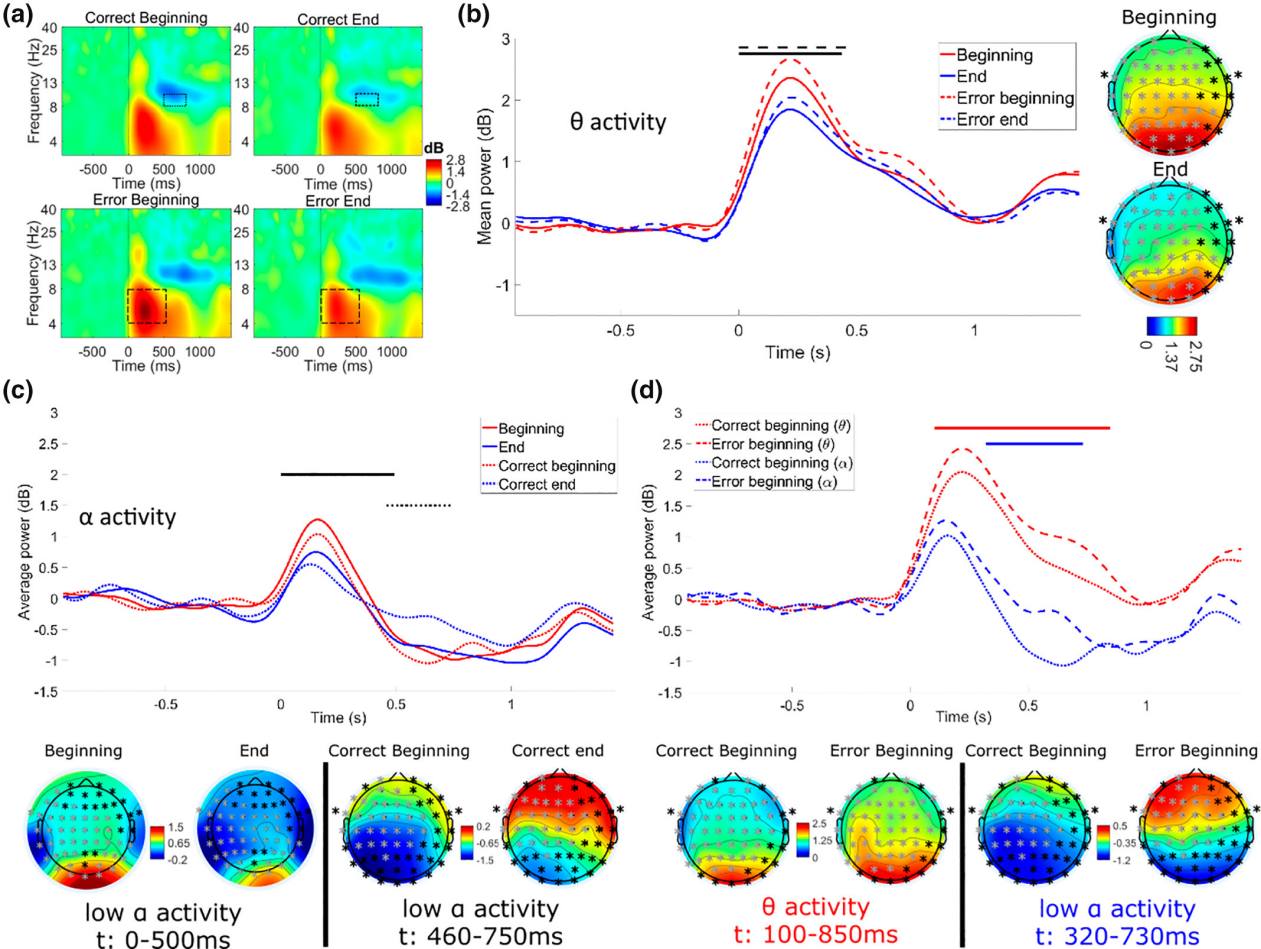
(a) Mean time‐frequency activity time‐locked to the system response display (0 ms), averaged across the 48‐electrode cluster for the four conditions (*accuracy* × *moment*) averaged across participants. Graphs show the power spectral density across trials (between ‐1 and 1.5 s) for each frequency (1‐40 Hz) according to baseline: higher values (increases) are in red and lower values (decreases) are in blue. The thin (upper panels – late alpha activity) and thick (lower panels – bheta activity) dotted lines display the outline of the significant clusters in terms of time and frequency windows. (b, c) Time course and topography of the clusters differentiating significantly power in the (b) theta and (c) alpha frequency bands at the beginning (red) and the end (blue) of the experiment for all responses averaged (plain line), erroneous responses (b – thick dotted line) and correct responses (c – thin dotted line). The black lines represent the significant approximate time‐window for the effect of the moment of the experiment for all responses averaged (b & c – plain line), for system errors only (b – thick dotted line) and for system correct response (c – thin dotted line). (b) Topographies show the average theta band power at the 48 electrodes of the cluster (gray crosses) during the significant 0‐440 ms time‐window (from 0 – blue – to 2.75 dB – red – relative to baseline) (c) topographies show the low alpha band power averaged across the 42‐electrode cluster (gray electrodes) in the 0‐500 ms significant time window for all responses – Left‐hand side – And over the 31‐electrode cluster (gray electrodes) in the 460‐750 ms significant time window for system correct responses – right‐hand side. (d) Time course and topographies of the significant difference in the theta (red, left‐hand side topographies) and low alpha (blue, right‐hand side topographies) frequency bands of the power spectral activity according to the accuracy of the system's response (correct responses – thin dotted lines – and errors – dashed lines) at the beginning of the experiment only. The black lines represent the significant time‐windows for the effect of system response accuracy on theta (red) and low alpha (blue) activities. Topographies show the average power spectral activity: in the theta frequency band at the 46 electrodes of the cluster (gray crosses) averaged across the 100‐850 ms significant time‐window – left‐hand side topographies – and in the low alpha frequency band at the 29 electrodes of the cluster (gray crosses) averaged across the 320‐730 ms significant time‐window – right‐hand side topographies.

Usual permutation analysis on time‐frequency data according to the accuracy and moment of the experiment for each frequency across all trials revealed no effect of any of these variables on the spectral power post‐system response at the FCz electrode. Nevertheless, as previously observed, visual inspection of the time‐frequency maps showed performance monitoring related activity through an increase of theta and low alpha activities compared to baseline in all conditions following system response observation, irrespective of its accuracy (see Figure 6a).

###### Theta activity (4‐8 Hz)

The cluster‐based permutation test revealed a significant difference in the theta frequency band (*p* < .01) between the end and the beginning of the experiment. This difference spanned over a time‐window from 0 to 440 ms after system response display and was more pronounced for all of the median and left‐lateralized electrodes (48 electrodes in total). A decreased power was observed at the end compared to the beginning, regardless the accuracy of system response. The time course and topography of this difference are presented in Figure 6b.

When looking at data according to the accuracy of system response, the cluster‐based permutation test revealed a decrease in the theta frequency band activity at the end compared to the beginning of the experiment for system error detection only (*p* < .05) extending approximately from 0 to 490 ms post‐system response and covering most of the scalp except for occipital electrodes (see Figure 6b).

Finally, at the beginning of the experiment only, the cluster‐based permutation test revealed a significant higher power in the theta frequency band after system error observation compared to correct response observation for a difference covering most of the scalp (46 electrodes) extending approximately between 100 and 850 ms post‐system response. This result is presented in Figure 6d.

###### Low alpha activity (8–10 Hz)

Regardless system response accuracy, the cluster‐based permutation revealed a significant difference in alpha frequency band power at the beginning compared to the end of the experiment for a large left‐lateralized centro‐parieto‐temporal cluster (42 electrodes) extending approximately from 0 to 500 ms post‐system response display (*p* < .05; see Figure 6c).

In a later processing time‐window, when considering correct responses only, the cluster‐based permutation test revealed another significant difference in left‐lateralized fronto‐centro‐temporal region (31 electrodes) spanning over a 460 to 750 ms post‐system response time‐window. The activity in the alpha frequency band was lower at the beginning compared to the end of the experiment (*p* < .05; see Figure 6c).

Finally, only at the beginning of the experiment, a difference in the left‐lateralized fronto‐centro‐temporal cluster (29 electrodes) related to system response accuracy was observed approximately from 320 to 730 ms post‐system response. The activity in the low alpha frequency band was significantly lower for observation of system correct responses compared to errors (*p* < .05; see Figure 6d).

## 4 DISCUSSION

The aim of this study was to investigate the expression of neural correlates reflecting performance monitoring, a cognitive mechanism whose electrophysiological features have been well‐defined in laboratory conditions, in a more dynamic and complex context. Indeed, several studies have demonstrated the difficulty to transpose results observed in standardized conditions to everyday‐life situations (Chavarriaga & Millán, 2010). At the same time, the study of performance monitoring and error detection during automated system supervision has become a major concern in various domains due to the increase in automation surrounding us (Chavarriaga et al., 2014; Ferrez & Millán, 2008). Here, we used a scenario adapted from the aeronautics field in order to better understand how brain activity evolves during system error monitoring over a long period of time, i.e., a very common situation in flight or during air traffic control. We recorded the EEG activity of 18 participants interacting with a conflict avoidance simulator (the Pilot‐System Interaction Lab – Gouraud et al., 2018; Le Goff et al., 2018). Two levels of task difficulty were considered (easy and difficult conditions) related to perceptual difficulty. Their influence on supervision‐related brain activity and the impact of the simulator response accuracy were measured. Then, we assessed how the moment of the experimental session modulates the supervision‐related brain activity according to the accuracy of system responses. Thanks to a robust statistical analysis with no a priori on the data (Maris & Oostenveld, 2007), we were able to observe an ERP differentiating between the cerebral activity related to correct and erroneous system response detection over a wide range of brain regions. In addition, we also observed a difference in the theta band activity in the time‐frequency domain in the trial time course bearing some similarities with performance monitoring activity observed in the literature (Cavanagh & Frank, 2014; Luu & Tucker, 2001). We observed, in this study, no effect of perceptual difficulty. However, we were able to highlight significant effects of the moment of the experiment on: (i) the low alpha activity, with a late increase at the end of the experiment compared to the beginning for correct system response; (ii) the early theta activity, with a decrease at the end of the experiment compared to the beginning both regardless system response accuracy and for system errors specifically. Overall, we evidenced activities related to performance monitoring and error detection, regardless task difficulty, associated with specific activities related to the supervision task and its length. We will now discuss our results according to these three axes.

### 4.1 Supervision activity: Errors vs. correct response detection

Concerning the supervision activity, several studies have identified a negative oERN component followed by a positive oPe, or an N2 peak followed by a P3 wave complex usually at later latencies (i.e., a frontocentral negative component followed by a centro‐parietal positive one) after another's error observation. Interestingly, this error‐observation‐related activity was found even in supervisory contexts without any movement or interaction required by the agent, may it be a human or an automated system (Somon et al., 2019b). The main issue addressed in this study was the evolution of such activity related to the observation of system's errors in a dynamic and complex context in terms of stimuli presentation. Even though the complexity of stimuli was increased, participants still demonstrated an overall very good ability to detect accurately system correct response and errors (average hit rate 97.6%) during the whole experiment (as demonstrated by the absence of difference on hit rates between the beginning and the end of the experiment).

In this supervisory context, we observed a significant difference in activity between error and correct response detection. This difference was significant on the whole scalp and was associated with a higher positive activity emerging between approximately 530 and 610 ms post‐system response for correct responses compared to error detection.

This component was not spatially specific. Difference topographies showed that, even though the whole scalp was involved, the greater activity for correct responses was mainly displayed at parieto‐occipital sites. Its more positive activity observed for correct responses could bear some similarities with a Reward positivity (RewP), a well‐known component observed during feedback monitoring whose role is to strengthen the link between a response and its result, after a positive feedback, through reinforcement learning (Fukushima & Hiraki, 2009; Holroyd & Coles, 2002; Proudfit, 2015). It has to be noted though that the RewP is generally observed during reward responsiveness paradigms, where a monetary reward is at stake (Proudfit, 2015). Our context of system supervision is a bit different, but the positive activity observed after correct response detection could correspond to the potentiation of the participant's estimated response, defined both by the processing of the stimulus they are performing and their confidence towards the system, by the accuracy of the system's response which is displayed. On another hand, the RewP signal bears similarities with the P300 wave. Yet, the P300 is known to be modulated by task difficulty both in terms of latency and amplitude (Leuthold & Sommer, 1998). At the latency level, several laboratory studies have demonstrated that the P300 latency is increased by the task difficulty, but also the quantity of stimulation noise surrounding the stimuli (Magliero et al., 1984). This effect has been demonstrated to be even stronger in demanding ecological tasks like operating a radar (Kramer et al., 1995) or an aircraft (Dehais et al., 2019). The present results provide novel evidence supporting this hypothesis and could provide more evidence that the RewP and P300 have, at least in part, common bases. Without formally naming this parieto‐occipital component, it shares several characteristics with usual performance monitoring ERPs. This component could emerge from the association of a negative fronto‐central and a wider positive centro‐parietal activity. In the context of performance monitoring activity, such a negative fronto‐central activity has been given different names – i.e., ERN, oERN, N2 – according to the situation at hand, and likewise for the positive activity – i.e., Pe, oPe, P300. The literature suggests that these two components would reflect the performance monitoring activity arising at different moment of cognitive control and decision making: whether before, during or after action selection and execution (Cavanagh & Frank, 2014; Somon et al., 2019b; Ullsperger, Fischer, et al., 2014). More broadly, the effects reported tend to support the theory of Predicted Response‐Outcome (PRO) according to which the performance monitoring activity is regulated at mediofrontal locations (by the pMFC) and corresponds to a feedback activity associated with learning on a Reward Prediction Error signal based on a response‐outcome pattern prediction (Alexander & Brown, 2010, 2011). Within this theoretical spectrum, supervising agents can learn from any type of outcome, without any motor response necessary, based on the reward prediction associated with that outcome. However, a better resolution of our ERPs in terms of number of trials would be required to confirm this hypothesis.

Compared to the evoked potentials and given the dynamic context of the task, the time‐frequency activity seems to be much more relevant and provides more information regarding the supervision‐related performance monitoring activity. The sensitivity of time‐frequency analyses to activities which are not phase‐locked or phase‐reset by the stimulus is much higher. Time‐frequency analyses made it possible to observe a cluster in the theta frequency band which was associated with an increased theta spectral activity between approximately 160 and 450 ms after system errors as compared to correct responses. It is interesting to note that error execution and detection has been associated with an increase in theta band activity on several occasions in laboratory standardized contexts (Cavanagh & Frank, 2014; Gehring et al., 2011; Luu & Tucker, 2001). This activity, though initially supposed phase‐locked (Luu et al., 2004), has been shown to be mostly non‐phase‐locked for both error execution, and error observation (Pezzetta et al., 2018). Our results on sorted trials time‐frequency analysis, as well as the coherence between the trials (ITC), support this hypothesis, given the low ITC values observed. Taken together, these results indicate that the theta band activity increase observed in our study is consistent with a performance monitoring activity taking place here during dynamic system supervision with complex stimulation. Concerning the spatial distribution of this theta activity provided by the topographies and difference topographies, it seems that there might be fronto‐temporo‐parietal theta involved in error detection compared to correct responses, but the link with usual Frontal‐Midline Theta (FMT) remains unclear as FCz and Cz electrodes do not seem to be involved in our study.

In addition, in the first half of the experiment only, the theta band activity difference was followed by a later lateralized fronto‐centro‐temporal alpha frequency difference discriminating significantly errors and correct responses detection. This significant difference revealed a lower alpha power from approximately 320 to 730 ms post‐response for correct responses compared to error detection. In the performance monitoring literature, a post‐error alpha suppression has been identified between roughly 300 and 500 ms post‐response in Simon and Sustained Attention to Response tasks (van Driel et al., 2012). Nevertheless, this post‐error alpha suppression is generally attributed to an attentional refocusing after a lapse (Carp et al., 2009; van Driel et al., 2012). In our study the errors are not the ones of the participant himself, thus do not demonstrate lapses in sustained attention, but errors of the system. It thus seems consistent that no reorienting alpha response should be observed. Moreover, a recent study about response observation in a continuous movement dynamic virtual reality context also displayed stronger alpha suppression (see Figure 5 in Pezzetta et al., 2018), here only assessed at the POz electrode, for avatar correct actions observation compared to error detection. In our case, latencies of this alpha suppression are similar, but the topographies tend to be more central. Finally, many studies have demonstrated a close relation between increase in alpha frequency band and hypovigilance in the literature (Borghini et al., 2014; Campagne et al., 2004; Craig et al., 2012), our results support a better vigilance and a better late processing for correct system responses compared to errors in the first half of the experiment.

To summarize, our study provides novel bricks for the characterization the activity related to error monitoring in a dynamic context where complex stimuli were presented. The results revealed that the spatio‐temporo‐frequential features of performance monitoring of dynamically evolving automated system had many similarities with the ones observed in the literature during execution tasks and supervision of another agent, although the executive and decision‐making processes involved are different in every situation. Nevertheless, two critiques remain on the more complex and dynamic aspect of our supervision task making it less realistic: (i) the recurrent freezing of the simulation which prevents from motor activity contamination on performance‐monitoring activity; and (ii) the high error rate of the simulator, allowing us to obtain enough trials in every condition for the statistical analyses. On this latter point, a study by Pezzetta et al. (2018) where they manipulated the error rate of a system during error observation, tends to show that performance monitoring activity (in the time and frequency domains) are not linked to error rates as they were still observable for an avatar performing 70% of errors. Still, several differences that we observed between correct system response and error detection in our study were not spatially specific, thus difficult to associate per se to the usual error monitoring activity.

### 4.2 Effect of task difficulty on performance supervision activity

A few studies in the literature have assessed the effect of task difficulty on performance monitoring activity but most of them were conducted in standardized lab environments with very simple stimuli. Both perceptual (Pailing & Segalowitz, 2004; Scheffers & Coles, 2000) and decisional difficulty (Van der Borght et al., 2016) have been shown to modulate some performance monitoring ERPs in execution and supervision tasks performed in laboratory conditions. As an illustration, an effect of task difficulty was reported for the N2 and the P3 associated with error detection during supervision task (Somon et al., 2019b). In more dynamic conditions though, this effect of the task difficulty on performance monitoring activity remains unclear. In our study, even though participants reported that the difficult condition was more difficult to supervise, no main effect of task difficulty was observed on neither their behavioral accuracy, nor the electrophysiological activity. The absence of effect on the behavioral data might come from the impact of the freezing of the simulation, providing participants enough time to make their decision, as well as the infinite time provided to them to respond. At the electrophysiological level though, one cluster was drawn from the data, discriminating between errors and correct responses. This cluster was associated with a positive potential whose characteristics tend to be similar to the RewP described in the previous paragraph and was only observed in the easy condition. One explanation could be the absence of such monitoring activity in the difficult condition. Yet, additional ERSP and ITC measures to assess performance monitoring activity appear more relevant than ERPs in our dynamic task. Despite a relative dynamic of performance monitoring‐related activities illustrated by a small inter‐trial coherence, a theta‐evoked ERSP activity related to accuracy of the system response was observed for both task difficulties. Another explanation suggested by the literature in task execution (Somon et al., 2019a; Van der Borght et al., 2016), proposes that overall perceptual difficulty increase in a task with complex stimuli taken from air‐traffic control simulations would decrease significantly the amplitude difference between error‐related and correct response‐related activities and could justify the absence of a significant cluster in the difficult condition. This effect associated with more restrictive (e.g., dynamical decision making, fewer data recorded) and varying recording conditions could blur the identification of the error‐detection activity in the difficult condition. In addition, the small number of trials in our experiment limits conclusions from ERP data.

Although the effect of task difficulty is weak in our study, it allows us to better decipher how performance monitoring processes unfold. The PRO theory (Alexander & Brown, 2010, 2011) supposes that the pMFC's response is maximal after an expected response not happening. In our study, correct responses from the system were more expected in the easy condition than in the difficult one. It follows that an error (i.e., the non‐occurrence of an expected correct response) triggered an increased activity in the easy condition compared to the difficult one. Thus, our results tend to support the PRO theory in dynamical and more complex contexts.

### 4.3 Effect of time on task on performance supervision activity

One major concern of our study was to determine the extent to which the performance monitoring activity would be degraded over time on task. Several studies have demonstrated a negative effect of time on task on performance supervision activity. Long and monotonous tasks are known to decrease vigilance and progressively reduce the ability to monitor correctly automated systems (i.e., the out‐of‐the‐loop phenomenon; Berberian, Gouraud, et al., 2017; Berberian, Somon, et al., 2017; Davies & Parasuraman, 1982; Matthews et al., 2002). However, their applied consequences on specific executive functions are not well documented.

In this study, even though no direct effect of the time on task on performances was observed, we showed from the time‐frequency data that performance monitoring activity really changes over time during an automated system supervision task. By comparing the cerebral activity in the first two blocks with the activity in the last two blocks of the experiment, we identified a significant difference of activity covering the major part of the scalp, even though slightly lateralized to the left, associated with a reduction of theta band spectral power with increasing time on task. When splitting error‐related from correct response‐related trials, this result was only observed when system errors were detected. The theta activity is usually linked to cognitive control and performance monitoring (Cavanagh & Frank, 2014; Luu & Tucker, 2001; Ullsperger, Danielmeier, & Jocham, 2014). In our case the decrease of this activity could reflect either a drop in error monitoring activity at the end of the experiment compared to the beginning, or a decreased ability to encode the information (Kutas et al., 1977; Luu & Tucker, 2001). Nevertheless, as mentioned earlier, it has to be noted that performance monitoring related cerebral activities can also be dissociated from performances themselves (Debener et al., 2005; Pezzetta et al., 2018). Thus, given the high error rate of the system in our experiment, the effect of time on task on theta activity could also be due to an habituation effect related to an initially high necessity to recruit cognitive resources to evaluate system responses, with this need decreasing over time.

In left fronto‐centro‐temporal regions, this theta activity was followed by another significant difference of activity associated with an increase of the alpha spectral power across time. Based on the literature (Borghini et al., 2014; Campagne et al., 2004), this increased alpha activity at the end of the experiment could reflect a degraded vigilance state or also a lower conscious perception of system responses. These results are consistent with the effect of time on task on alpha activity usually studied in the case of execution tasks. In addition, in our supervision task, we showed a lower alpha activity after correct response observation compared to error detection at the beginning of the experiment only. This result could be explained by either an impact of confidence or of consciousness towards the stimulus. Indeed, some studies have shown that attention orientation, especially to task relevant stimuli, and conscious perception of stimuli could produce an alpha‐amplitude reduction (Babiloni et al., 2006; Harris et al., 2017). In a no‐report inattentional blindness paradigm using cluster‐based permutation analyses, Harris et al. (2018) showed a significant decrease in post‐stimulus contralateral alpha power related to the awareness of probe stimuli. Our analysis however, only included trials (for errors and correct responses) to which the participants responded to with accuracy. Consequently, we should not expect any difference in system response awareness, and no specificity of the moment of the experiment. One the other hand, our study permitted to highlight physiological markers associated with the effect of time on task on the degradation of the monitoring activity (i.e., the decrease in theta activity and increase in alpha activity). Here, the cause of such degradation cannot be identified. Several causes are known to give rise to effects on psychophysiological activity according to the time spent supervising automated systems. Namely monotony, fatigue, hypovigilance, loss of agency or increased complacency towards automated systems have been demonstrated as causing a decreased supervision activity. Taking the example of trust, trust towards the automated system or confidence towards its responses, can vary over time. Behavioral as well as EEG experiments revealed an attentional bias towards automated systems, with an initial over‐confidence due to an automation bias leading participants to consider automation as being reliable (de Visser et al., 2018; Parasuraman & Manzey, 2010), followed by an updating of trust through reinforcement learning across time (Goodyear et al., 2017). This updating can build up confidence towards the system response and thus in the forthcoming response of the participant. An experiment (Gherman & Philiastides, 2015) showed that brain activity associated with participants' confidence at the single trial level was building up starting from 300 ms and peaking 600 ms post‐stimulus, as discrimination increased gradually between certain and uncertain trials. Likewise, Desender et al. (2019) observed in a two‐time choice‐response task that neural processes could link confidence towards one's decision to observation seeking behavior at the post‐decisional timescale. In our experiment, late post‐stimulus alpha could show confidence building towards the system's response. In this pattern, confidence would both be impacted by the type of response, requiring more information seeking for errors in order to ensure that response, but also by the moment of the experiment. Indeed, the system's accuracy level was unknown at the beginning of the experiment by all participants, who could have learned it and have a better error expectation by the end. Lower alpha activity for correct response observation compared to error detection at the beginning of the experiment would thus illustrate a higher confidence in correct response detection (and, conversely for error detection), which could decrease with time on task, as illustrated by a higher alpha activity at the end the experiment. Such a decrease of overall trust towards the automated system during system monitoring between pre‐ and post‐experiment has already been observed (Goodyear et al., 2017). Still, trust towards the automated system and resulting complacency are not the only cause of operator disengagement in system supervision and being able to precisely identify the neurophysiological correlates of said disengagement could allow us to lean towards one cause or another.

## 5 CONCLUSION AND PERSPECTIVES

In this study we provided new insights into performance monitoring‐related brain activities during supervision of an automated system in more dynamic situations where complex stimuli were presented as compared to usual lab tasks. We showed similarities with results obtained in more standardized laboratory conditions. Importantly, our results reveal a degradation of monitoring activity with increasing time on task, which could potentially constitute a biomarker reflecting an inability to monitor correctly the automated systems (i.e. the out‐of‐the‐loop phenomenon). Finally, we demonstrate the relevance of time‐frequency analyses in dynamic contexts, and the need to systematically perform ERSPs in addition to ERPs as the former contain richer information. Taken together, these different insights open interesting avenues regarding the online detection of such neural markers and their degradation as operators' complacency towards automated systems increases. Given the increasing automation of systems and the difficulties traditionally observed during their supervision, these advances reflect an undeniable tool for improving safety in several areas where human operators are reduced to supervisory control.

What makes automation particularly detrimental to the operator's sense of agency is not yet fully understood, but there is a relative consensus that the lack of transparency on how the system makes its decisions, or simply operates, is a key factor (Christoffersen & Woods, 2002; Klien et al., 2004; Norman, 1990).

## AUTHOR CONTRIBUTIONS

**Bertille Somon:** Conceptualization; data curation; formal analysis; investigation; methodology; project administration; software; visualization; writing – original draft; writing – review and editing. **Aurélie Campagne:** Conceptualization; methodology; project administration; resources; software; supervision; validation; writing – review and editing. **Arnaud Delorme:** Formal analysis; software; supervision; validation; writing – review and editing. **Bruno Berberian:** Conceptualization; funding acquisition; methodology; project administration; resources; supervision; validation; writing – review and editing.

## FUNDING INFORMATION

This work was supported by the ‘Région Provence‐Alpes‐Côte d'Azur’ (Emploi Jeunes Doctorants – 2015_07459) and the ANR/FRAE (Young researcher program – ANR‐15‐CE26‐0010‐01).

## CONFLICT OF INTEREST

The authors declare that the research was conducted in the absence of any commercial or financial relationships that could be construed as a potential conflict of interest.

## REPRINTS

Requests for reprints should be sent to Bertille Somon (bertille.somon@onera.fr).

## Supporting information


**Figure S1** Trial‐by‐trial time‐frequency of theta activity (ERP‐Image – top) and inter‐trial phase coherence (ITC – bottom) averaged across participants at the FCz electrode time‐locked to the system's response display (0ms) according to (a and c) accuracy (system correct responses – top – and errors – bottom) and task difficulty (difficult condition – left – and easy condition – right) and (b and d) accuracy (system correct responses – top – and errors – bottom) and moment of the experiment (beginning of the experiment – left – and end of the experiment – right). ERP‐images show the activity trial‐by‐trial sorted according to the maximum of the phase time‐locked to the theta activity peak latency (i.e., 500 ms after system response). Positive values (red) and negative values (blue) are relative to baseline. ITC display phase consistency over trials for all frequencies (1–40 Hz). Higher values (red) show a better synchronization of phases to the time‐locking event (system response) as opposed to lower (green) values

## Footnotes

^1^
Another baseline from −1000 to −500 ms was tested with similar results.
