# Supplementary figures and images for: Brain mechanisms of automated conflict avoidance simulator supervision
Source: Psychophysiology. 2022 Sep 15;60(2):e14171. doi: 10.1111/psyp.14171 (PMC10078105; doi:10.1111/psyp.14171)

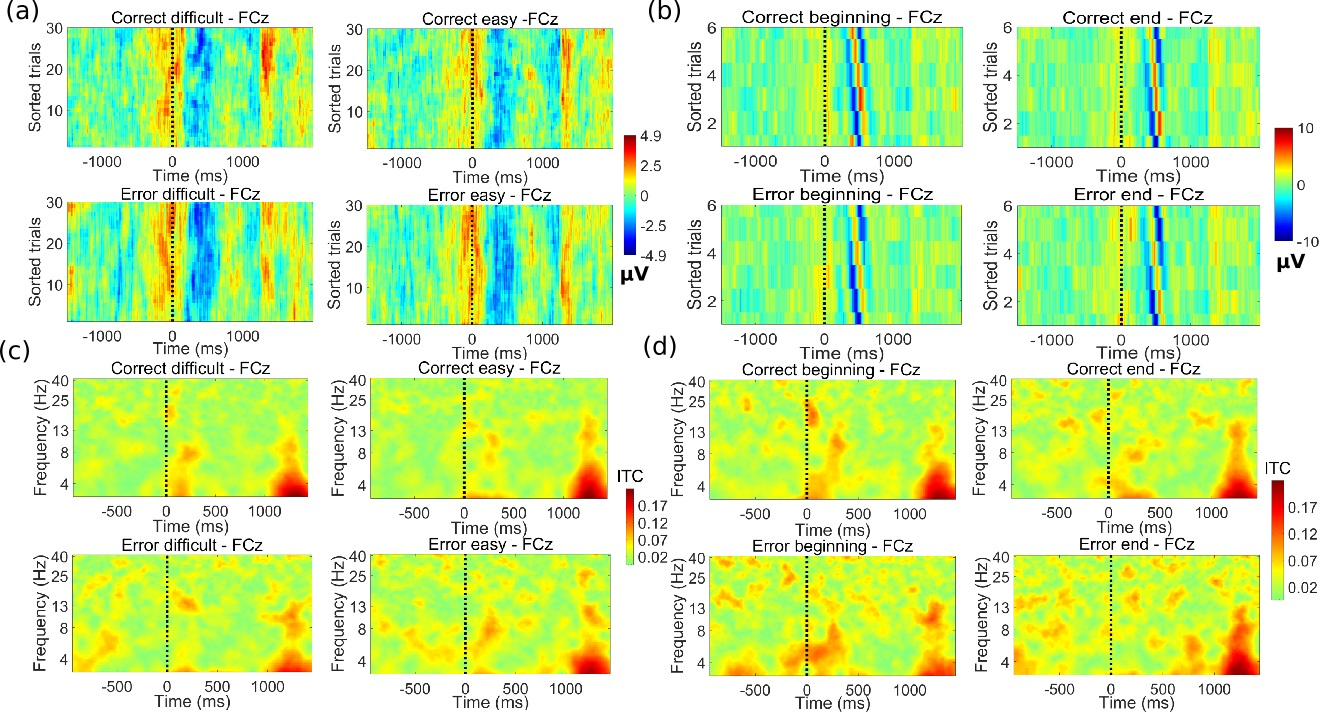

Supplement: Supplementary file 1 — Figure S1 Trial‐by‐trial time‐frequency of theta activity (ERP‐Image – top) and inter‐trial phase coherence (ITC – bottom) averaged across participants at the FCz electrode time‐locked to the system's response display (0ms) according to (a and c) accuracy (system correct responses – top – and errors – bottom) and task difficulty (difficult condition – left – and easy condition – right) and (b and d) accuracy (system correct responses – top – and errors – bottom) and moment of the experiment (beginning of the experiment – left – and end of the experiment – right). ERP‐images show the activity trial‐by‐trial sorted according to the maximum of the phase time‐locked to the theta activity peak latency (i.e., 500 ms after system response). Positive values (red) and negative values (blue) are relative to baseline. ITC display phase consistency over trials for all frequencies (1–40 Hz). Higher values (red) show a better synchronization of phases to the time‐locking event (system response) as opposed to lower (green) values [file PSYP-60-0-s001.png]
