# Supplementary material for: Brain mechanisms of automated conflict avoidance simulator supervision
Source: Psychophysiology. 2022 Sep 15;60(2):e14171. doi: 10.1111/psyp.14171 (PMC10078105; doi:10.1111/psyp.14171)
Supplement: Supplementary file 2 [file PSYP-60-0-s002.docx]

Supplementary materials

**Trial-by-trial time-frequency analysis and inter-trial phase coherence**

In order to assess the temporal dynamics of system response evaluation and variance across trials in the conflict avoidance simulator supervision task, trial-by-trial time-frequency and ITC analyses were performed in the theta frequency band which is usually linked to decision making. For ERP-images, trials were sorted according to the maximum of the phase time-locked to the theta activity peak latency.

Here, we show the results time-locked to the system’s response display (t=0ms) in the various experimental conditions with the two different statistical analyses: 1) with accuracy and task difficulty as factors, and 2) with accuracy and moment of the experiment as factors.

1. Analysis according to accuracy and task difficulty

No significant difference in trial-by-trial time-frequency analysis (ERP-image) of the theta activity was observed between the four experimental conditions. Nevertheless, the temporal dynamic of the trial-by-trial theta response, locked to its phase maximum at the peak latency suggests a theta activity phase-locked to the system response display, i.e. an evoked theta activity, rather than induced (figure S1a). Here, ITC values were lower than 0.2 (figure S1c).

1. Analysis according to accuracy and moment of the experiment

There was no effect of the accuracy, nor of the moment of the experiment, on the ERP-images (figure S1b).

Similarly to the first analysis, ITC values were lower than 0.2 (figure S1d).

**--- INSERT FIGURE S1 ---**

**Figure S1.** Trial-by-trial time-frequency of theta activity (ERP-Image - top) and inter-trial phase coherence (ITC - bottom) averaged across participants at the FCz electrode time-locked to the system’s response display (0ms) according to (a and c) accuracy (system correct responses – top – and errors – bottom) and task difficulty (difficult condition – left – and easy condition – right) and (b and d) accuracy (system correct responses – top – and errors – bottom) and moment of the experiment (beginning of the experiment – left – and end of the experiment – right). ERP-images show the activity trial-by-trial sorted according to the maximum of the phase time-locked to the theta activity peak latency (i.e., 500ms after system response). Positive values (red) and negative values (blue) are relative to baseline. ITC display phase consistency over trials for all frequencies (1-40Hz). Higher values (red) show a better synchronization of phases to the time-locking event (system response) as opposed to lower (green) values.
